# The evolutionary origins of pesticide resistance

**DOI:** 10.1111/brv.12440

**Published:** 2018-07-03

**Authors:** Nichola J. Hawkins, Chris Bass, Andrea Dixon, Paul Neve

**Affiliations:** ^1^ Department of Biointeractions and Crop Protection Rothamsted Research Harpenden AL5 4SE U.K.; ^2^ Department of Biosciences University of Exeter, Penryn Campus Cornwall TR10 9FE U.K.; ^3^ Department of Plant Biology University of Georgia Athens GA 30602 U.S.A.

**Keywords:** evolution, pesticide resistance, herbicide, fungicide, insecticide, standing variation, *de novo* mutation, adaptive introgression, pleiotropic co‐option, selective sweeps

## Abstract

Durable crop protection is an essential component of current and future food security. However, the effectiveness of pesticides is threatened by the evolution of resistant pathogens, weeds and insect pests. Pesticides are mostly novel synthetic compounds, and yet target species are often able to evolve resistance soon after a new compound is introduced. Therefore, pesticide resistance provides an interesting case of rapid evolution under strong selective pressures, which can be used to address fundamental questions concerning the evolutionary origins of adaptations to novel conditions. We ask: (*i*) whether this adaptive potential originates mainly from *de novo* mutations or from standing variation; (*ii*) which pre‐existing traits could form the basis of resistance adaptations; and (*iii*) whether recurrence of resistance mechanisms among species results from interbreeding and horizontal gene transfer or from independent parallel evolution. We compare and contrast the three major pesticide groups: insecticides, herbicides and fungicides. Whilst resistance to these three agrochemical classes is to some extent united by the common evolutionary forces at play, there are also important differences. Fungicide resistance appears to evolve, in most cases, by *de novo* point mutations in the target‐site encoding genes; herbicide resistance often evolves through selection of polygenic metabolic resistance from standing variation; and insecticide resistance evolves through a combination of standing variation and *de novo* mutations in the target site or major metabolic resistance genes. This has practical implications for resistance risk assessment and management, and lessons learnt from pesticide resistance should be applied in the deployment of novel, non‐chemical pest‐control methods.

## I. INTRODUCTION: EVOLUTION OF PESTICIDE RESISTANCE

As the world anticipates feeding nine billion people as sustainably as possible by 2050, crop protection against pest insects, diseases and weeds has a vital role in maintaining and improving crop yields (Godfray *et al.,* 2010). Whilst awareness is growing of the importance of integrated pest management, pesticides remain a necessary part of the pest‐control toolbox for many crops (Swanton *et al.,* 2011), in combination with other approaches such as disease‐resistant crop varieties (Carolan *et al.,* 2017). However, just as the effectiveness of antibiotics in the control of human disease is under threat due to the evolution of resistant strains of bacteria (World Health Organisation, 2014), the control of agricultural pests and crop diseases is threatened by the evolution of pesticide resistance, affecting insecticides (Bass *et al.,* 2015), herbicides (Powles & Yu, 2010) and fungicides (Lucas, Hawkins & Fraaije, 2015). Modern pesticides aim for specificity to reduce non‐target effects in the environment (Vyas, 1988), but this specificity also means that resistance is more evolutionarily accessible to the intended target pests.

Most studies of pesticide resistance focus on the proximate biochemical mechanism conferring reduced sensitivity to the compound in question, whether by target‐site mutations or over‐expression, or metabolic breakdown or efflux of the pesticide. However, the emergence and spread of resistance are evolutionary processes, and greater understanding of the evolutionary mechanisms involved can inform resistance risk assessment and management strategies (Maclean *et al.,* 2010; Neve *et al.,* 2014). Furthermore, pesticide resistance is a key example of evolution in action, with rapid evolution under novel selective pressures (Palumbi, 2001), and has the potential to contribute to fundamental understanding of general evolutionary processes. Resistance could be considered as an example of evolutionary rescue (Alexander *et al.,* 2014), and could address questions concerning adaptation to changing environments, sources of variation and origins of novel traits.

In this review, we consider what is known about the evolutionary origins of resistance to pesticides, and where further studies are needed. We focus on the relative importance of *de novo* mutations and standing variation, the role of interspecific gene transfer, and pre‐adaptation through pleiotropic effects of existing adaptations.

### (1) *De novo* mutations

**Figure 1:**
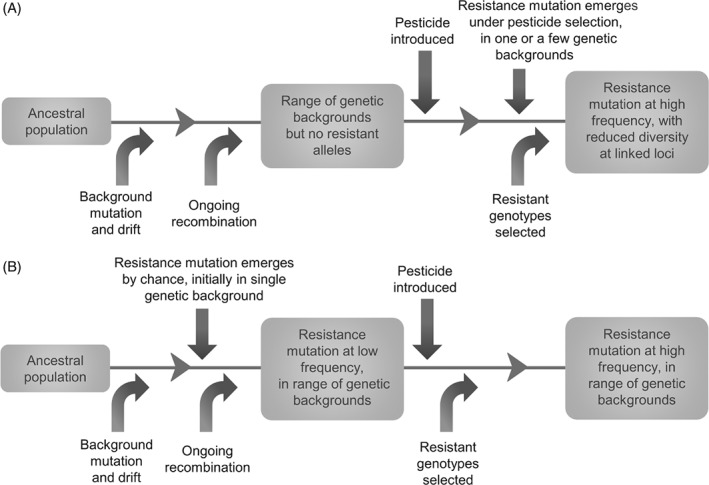
Evolution of pesticide resistance: (A) by *de novo* mutation; (B) by selection from standing genetic variation.

In evolutionary terms, a *de novo* mutation is one which originates once an environmental change has occurred making that mutation selectively advantageous (Messer & Petrov, 2013), and emerges under that selection. In the case of pesticide resistance, this would mean a mutation conferring resistance occurs after the introduction of the pesticide (Fig. 1A). A mutation may arise once and spread through the population, or there may be multiple, independent *de novo* origins.

A small number of studies have implicated possible mutagenic effects of β‐lactam antibiotics (Gutierrez *et al.,* 2013) and quinone outside inhibitor (QoI) fungicides (Schnabel & Chen, 2013; Chen *et al.,* 2015), and speculated possible similar effects for herbicides (Gressel, 2011). However, the general assumption for *de novo* resistance evolution is that mutation continues at the background rate, but strong selection by the pesticide means that mutations conferring resistance will increase in frequency, rather than mostly being lost through genetic drift.

### (2) Standing variation

In the case of selection from standing variation, polymorphisms are already present in the population prior to the change in selective pressure (Barrett & Schluter, 2008), whether in a single gene or a polygenic trait (Pritchard & Di Rienzo, 2010). Following the environmental change, a pre‐existing neutral or deleterious allele becomes advantageous and is selected to a higher frequency in the population (Fig. 1B).

Cryptic variation (Gibson & Dworkin, 2004) could include polymorphisms in genes that are only highly expressed in resistant individuals (Rajon & Masel, 2013), or variation interacting epistatically with resistance mutations, such as compensatory mutations increasing the stability of mutant proteins (Cools *et al.,* 2010). Therefore, even when resistance itself emerges through a *de novo* mutation, standing variation in genetic background may affect the fitness of resistant individuals.

### (3) Intrinsic resistance and pleiotropic co‐option

Standing variation means that resistant alleles are already present, but at low frequencies prior to pesticide selection. However, some species have a degree of pre‐existing resistance already fixed in the population prior to pesticide exposure. In some cases, the level of pre‐existing resistance is high enough that the species is considered intrinsically resistant (Lucas *et al.,* 2015). In other cases, pre‐existing adaptations such as the efflux or metabolism of naturally occurring toxins are insufficient to confer intrinsic high‐level resistance to pesticides, but once under selection for their pleiotropic effects on pesticide resistance, they may evolve increased activity, for example by overexpression (Kretschmer *et al.,* 2009) or gene amplification (Mouches *et al.,* 1986). Such over‐expression or amplification may originate through *de novo* mutations, including transposons or duplications, or be selected from standing variation. In this scenario, a pre‐existing adaptation is co‐opted as a resistance mechanism, and pesticide resistance, previously a co‐incidental pleiotropic effect, becomes the major selective force on the further evolution of that trait. This pre‐existing resistance may be due to exposure to similar, naturally occurring compounds (Bass *et al.,* 2013), or to pleiotropic effects of non‐xenobiotic‐related adaptations (Song *et al.,* 2011): this is discussed in more detail in Section III.

### (4) Interspecific transfer

**Figure 2:**
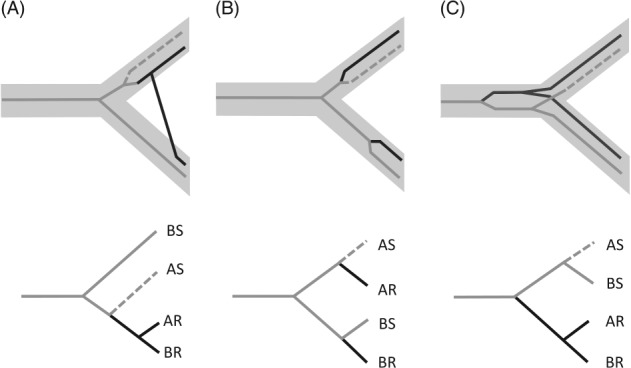
Phylogenetic origins of pesticide‐resistant alleles. The upper part of each panel shows genes superimposed onto a species tree; the lower part shows the resulting gene tree. (A) Allele transfer by horizontal gene transfer or adaptive introgression; (B) parallel evolution by independent *de novo* mutations; (C) collateral selection from pre‐speciation standing variation. AS: species A, sensitive allele; BS: species B, sensitive allele; AR: species A, resistant allele; BR: species B, resistant allele. Grey lines, sensitive allele; black lines, resistant allele; dashed lines, sensitive allele may have been lost from sister species if resistance has previously reached fixation.

Where pesticide resistance (intrinsic or acquired) is present in one species, the resistance allele may sometimes move into other, target pest species, through interbreeding or by horizontal gene transfer (HGT) (Fig. 2A). Alternatively, occurrence of the same resistant allele in different species may be the result of parallel evolution, for genetic changes that are simple enough to have arisen independently in multiple lineages (Fig. 2B); or, in sister species, shared resistant alleles may be the result of collateral selection, whereby the sensitive and resistant alleles diverged before the species separated, followed by selection of the common resistant allele in both species (Fig. 2C) (Stern, 2013).

Interbreeding may include adaptive introgression, in which hybridisation between a ‘donor’ species and a target species is followed by back‐crosses with the target species during which positive selection results in the retention of a specific gene from the donor species (Hedrick, 2013). For example, the house mouse *Mus musculus domesticus* has gained rodenticide resistance alleles through hybridisation with the intrinsically resistant Algerian mouse *Mus spretus*, followed by introgression under rodenticide selection (Song *et al.,* 2011; Liu *et al.,* 2015).

HGT is most common in bacteria, to the extent that for plasmid‐borne antibiotic resistance the source population for standing variation is a multi‐species ‘resistome’ (Forsberg *et al.,* 2012). However, HGT has also been reported in some eukaryotes, including fungi (Friesen *et al.,* 2006).

## II. PRACTICAL IMPLICATIONS

### (1) Repeatability

Whilst the repeatability of evolution is a long‐standing fundamental question in evolutionary biology (Lässig, Mustonen & Walczak, 2017), when applied to resistance it also has important practical implications. Molecular diagnostics can be developed for the rapid detection of resistant alleles, but the reliability of such diagnostics depends upon the likelihood that different populations have evolved the same or different resistance mechanisms. Furthermore, the applicability of management strategies across different pest populations may depend on whether the same resistance mechanisms are evolving in each case.

Experimental evolution studies suggest that *de novo* mutations show low repeatability, with replicate populations showing phenotypic but not genotypic convergence (Bedhomme, Lafforgue & Elena, 2013), whereas selection from standing variation is more repeatable (Burke, Liti & Long, 2014) since collateral evolution can take place by selection from the same allele pool (Stern, 2013) without the need for random new mutations within each population.

However, pesticide use exerts an exceptionally specific selective pressure, especially in cases of target‐site resistance where selection acts not just on a single trait but often on a single molecular target and even specific pesticide‐binding residues, with further constraints imposed by the need to maintain the original function of the target protein (Cools, Hawkins & Fraaije, 2013). This can result in parallel *de novo* mutations, such as G143A in cytochrome *b*, responsible for most cases of resistance to QoI fungicides (Grasso *et al.,* 2006), and A302S in *Rdl*, associated with resistance to cyclodiene insecticides in multiple insect orders (Thompson, Steichen & Ffrench‐Constant, 1993). By contrast, the complex constraints and epistatic interactions shown by CYP51 mean that azole resistance has evolved through a wide range of different target‐site mutations (Cools *et al.,* 2013).

Furthermore, experimental evolution studies into selection from standing variation use replicate populations with the same initial genotype mix (Burke *et al.,* 2014), but field populations may have different initial gene pools. Populations that have undergone bottlenecks will have lost some of the initial standing variants, whereas populations that have been isolated for a long time may have independently accumulated subsequent unique mutations into standing variation.

Therefore, the repeatability of selection from common standing variation depends upon the degree of divergence between populations, whereas the repeatability of *de novo* mutations depends on the functional constraints affecting each target site and other possible resistance mechanisms. In the case of interspecific gene transfer, introgression from a known source such as a herbicide‐resistant crop would be more repeatable than independent evolution of resistance within pest species, whereas introgression from a wider pool of wild relatives, or HGT from more distantly related species, would be less repeatable.

### (2) Probability and speed of emergence

Standing variation comprises alleles already present in the population, whereas for *de novo* mutations there will be some waiting time for suitable mutations to occur (mutation limitation), depending on population size and mutation rate (Karasov, Messer & Petrov, 2010). Therefore, resistance could be expected to emerge more rapidly if resistant alleles already exist within the standing variation; whereas for *de novo* mutations, initial emergence as well as subsequent selection of mutations should be considered in resistance management strategies (Hobbelen, Paveley & Van Den Bosch, 2014).

Furthermore, a *de novo* mutation starts as a single copy, whereas alleles present as standing variation will already be present in multiple individuals (Hermisson & Pennings, 2005). Since the probability of loss or fixation of a resistance allele depends on both the initial frequency and the selection coefficient, a single‐copy *de novo* mutation would only be likely to emerge if it had a high positive selection coefficient, whereas a standing variant already present at a higher frequency may increase in frequency even with a weaker selective benefit. For pesticide resistance, the selection coefficient is higher for mechanisms conferring greater levels of resistance, but reduced by any associated fitness costs. Therefore, resistance mechanisms conferring high resistance factors can emerge from *de novo* mutations, whereas mechanisms conferring only partial resistance, or carrying significant fitness penalties, are more likely to emerge from standing variation. The emergence risk of partial rather than complete resistance has implications for the optimal dose rates for resistance management (Van Den Bosch *et al.,* 2011).

Similarly, in diploid and polyploid species, a recessive allele is less likely to arise *de novo* or by hybridisation, since a new gene would initially be heterozygous, whereas a standing variant may already have reached sufficient frequency for homozygotes to be present in a recombining population so selection is less dependent on genetic dominance (Orr & Betancourt, 2001). Introgressed genes would also be heterozygous in the F1 generation, with further crosses, or backcrosses with other hybrid progeny, required before a recessive allele could confer a fitness effect, so emergence probability would depend on dominance as well as hybridisation frequency.

The risk of resistance emerging through pleiotropic co‐option depends on the presence of suitable efflux pumps or metabolic pathways, and the evolutionary distance between their initial state and acquired resistance to the pesticide. There may be a greater risk of a suitable efflux pump or metabolic pathway being available in species dealing with a wider range of natural toxins, but structure–substrate relationships are not sufficiently well understood to predict which specific efflux or metabolic genes may contribute to resistance risk for a given pesticide.

### (3) Co‐adaptation and complex traits

Whilst *de novo* adaptations to sudden environmental changes must emerge rapidly, standing variation may have accumulated over a longer time scale, allowing rarer genetic changes to occur and more complex traits to evolve. It has also been argued that complex genetic changes are more likely to originate as cryptic variation, since less‐fit intermediate states would not be exposed to negative selection (Rajon & Masel, 2013).

In the case of interspecific transfer from an intrinsically resistant species, the longer timescales over which resistance has evolved in the source species also allow rare or multiple genetic changes to occur, but wider genomic co‐adaptation may be lost with crossing or with the horizontal transfer of a small genetic region.

### (4) Spread of resistance

A *de novo* mutation with a single origin may spread through the movement of insects, seeds and pollen, or spores, whereas an allele present in standing variation may already be present throughout the range of a pest if it arose prior to the spread of the pest species to different regions. Therefore, the origin of resistance can determine the most effective containment strategy. Where a *de novo* resistance mutation is present in a limited area, quarantine measures could limit its spread, whereas if resistance is already present within standing variation across a pest's range, resistance management to prevent parallel selection within each region is more important. For interspecific origins, a resistant strain originating from a rare HGT, or a hybrid with a relative with a restricted range, should be treated more like a *de novo* mutation, with quarantine measures considered, whereas crosses with a widespread relative or the crop itself are more likely to recur independently in other regions and efforts should be focussed on resistance‐management practices that reduce local selection.

A major current debate concerning the origin of resistance is centred on azole resistance in the soil‐dwelling saprophyte and opportunistic clinical pathogen *Aspergillus fumigatus*. Resistance to clinical azole anti‐fungal drugs is a growing problem, and research is ongoing to determine the origin of resistance in previously untreated patients. The occurrence of specific combinations of coding mutations and promoter inserts points to selection from standing variation in inoculum rather than *de novo* mutations within each patient (Verweij *et al.,* 2013), but the origins of those resistant genotypes in the environmental population, and whether they have been selected by agricultural or other uses of azole fungicides, are not yet clear (Gisi, 2014).

In the case of genetically modified or mutagenised herbicide‐resistant crops, resistant weeds may evolve due to gene flow from the crop, or from independent parallel evolution within the weed species (whether from *de novo* mutations or standing variation within the weed population). This has implications for whether genetic modification itself is culpable for the evolution of ‘superweeds’, or whether it is purely the result of associated agronomic practices involving extensive repeated use of a single herbicide mode of action. Risk assessments for the release of new herbicide‐resistant crops consider the presence of related weed species in areas where the crop will be grown, but growing evidence that most ‘superweeds’ result from parallel selection rather than hybridisation (see Section V.2) indicates that agronomic risk factors and resistance‐management guidelines are more important.

### (5) Genomic studies

Next‐generation high‐throughput sequencing methods are becoming increasingly important tools in resistance studies, especially for non‐target‐site mechanisms (e.g. Van Leeuwen *et al.,* 2012; Gaines *et al.,* 2014; Omrane *et al.,* 2015). Population genomic analyses can identify genes under selection. New methods are being developed to detect selective sweeps that are currently underway before the selected gene has reached a high frequency in the population, but the success of these methods depends on whether the selective sweep underway is a hard sweep from a single *de novo* origin or a softer sweep from multiple or standing mutations.

Resistance studies more commonly look for genomic or transcriptional differences correlated with resistant phenotypes (e.g. Omrane *et al.,* 2015). The evolutionary origin of resistance mutations is also relevant to the design of these studies: where a resistance mechanism originates from a single *de novo* mutation with a recent hard selective sweep, hitchhiking genes may also appear to be correlated with resistance, so it would be preferable to carry out crosses if possible and sequence the progeny rather than field strains, through a bulk segregant approach as used in previous random amplification of polymorphic DNA (RAPD) marker studies (Fabritius, Shattock & Judelson, 1997), or a quantitative trait locus (QTL) approach (Lendenmann, Croll & Mcdonald, 2015). Where a resistance mechanism has been selected from standing variation in an outcrossing species, field isolates will already have undergone extensive recombination, so additional resistance‐correlated genes may point to intragenomic co‐adaptation, with those genes conferring a selective advantage in the presence of the primary resistance allele.

### (6) Design of selection experiments and baseline monitoring

Evolutionary researchers have considered the relative roles of *de novo* mutations and standing variation in experimental evolution (Burke *et al.,* 2014). However, for pesticide resistance, selection experiments also have practical significance in attempting to predict the future evolution of resistance to new pesticides.

Selection experiments from a single parental isolate investigate the potential for *de novo* mutations, since there is no standing variation, and may use ultraviolet (UV) or chemical mutagenesis to increase the mutation rate. Scalliet *et al*. (2012), Fraaije *et al*. (2012) and Gutiérrez‐Alonso *et al*. (2017) used *in vitro* selection of individual fungal isolates with UV mutagenesis to explore possible mutations conferring resistance to newly developed succinate dehydrogenase inhibitor (SDHI) fungicides. This approach was appropriate given the apparent prevalence of *de novo* point mutations in target‐site resistance to fungicides, and the sdhC‐H152R substitution generated by Scalliet *et al*. (2012) has subsequently been reported in the field (Dooley *et al.,* 2016). However, UV mutagenesis did not predict the emergence of field strains with slightly reduced SDHI sensitivity due to a non‐target‐site mechanism comprising overexpression of the major facilitator efflux pump MgMfs1 (Omrane *et al.,* 2015).

Lagator, Colegrave & Neve (2014) and Lagator *et al*. (2013) used the alga *Chlamydomonas reinhardtii* as a laboratory model organism for the experimental evolution of herbicide resistance, investigating the effects of alternating or mixing different modes of action. The parental strain was isolated from a single‐cell colony, and *C. reinhardtii* is asexual in laboratory conditions; therefore, all differences in herbicide sensitivity must have arisen *de novo* during the experiment. This may affect the likelihood of strains emerging with simultaneous resistance to multiple herbicides.

Selection experiments with chemical mutagenesis in the model insect species *Drosophila melanogaster* have been used to identify or confirm target sites of neonicotinoids, spinosyns and methoprene, but their accuracy in predicting which mutations will arise in field populations of pest insects has been variable (Shemshedini & Wilson, 1990; Perry *et al.,* 2008; Watson *et al.,* 2010). However, mutagenesis and selection of laboratory populations of Australian sheep blowfly, *Lucilia cuprina*, have successfully reproduced insecticide‐resistance alleles previously detected in the field, including target‐site resistance to dieldrin and major‐gene metabolic resistance to organophosphates (McKenzie & Batterham, 1998). In this case, the metabolic resistance was conferred by gain‐of‐function single‐nucleotide polymorphisms (SNPs), which could readily arise *de novo*.

In contrast to such *in vitro* mutagenesis studies, resistance risk assessment through the screening of field populations to detect outliers from the baseline sensitivity distribution (Russell, 2004) investigates the presence of reduced sensitivity in standing variation (Espeby, Fogelfors & Milberg, 2011; Ulber, Nordmeyer & Zwerger, 2013).

## III. PRE‐PESTICIDE ORIGINS OF RESISTANCE

Where resistance pre‐dates pesticide selection, either within standing variation or fixed as intrinsic resistance, this may be due to selection for resistance to naturally occurring toxins, but it may also be due to pleiotropic effects of a non‐resistance‐related adaptation, or the chance results of neutral processes and not adaptive at all until the introduction of the pesticide.

### (1) Natural toxins

In the case of antibiotic resistance, many compounds were originally derived from allelopathic products of soil microbes, so selection for resistance in natural antibiotic producers and neighbouring bacteria pre‐dates anthropogenic antibiotic use (Perry & Wright, 2013). The strobilurin fungicides are also based on natural allelopathic compounds, produced by species of the basidiomycete fungi *Strobilurus*, *Mycena* and *Oudemansiella*, which are self‐resistant due to cytochrome *b* mutations (Kraiczy *et al.,* 1996), including the G143A amino acid substitution responsible for acquired strobilurin resistance in many phytopathogenic fungi (Zheng, Olaya & Koller, 2000). However, most plant pathogens are unlikely to have evolved in frequent contact with strobilurin‐producing fungi, so the simple nature of strobilurin resistance makes it more likely that acquired resistance in target species evolved through multiple parallel *de novo* origins of similar mutations.

It is highly improbable that more‐complex toxin‐resistance mechanisms, such as efflux transporters and metabolic detoxification enzymes, have arisen *de novo* within the short time for which pesticides have been used: the existence of transporters and detoxification enzymes pre‐dates anthropogenic pesticide use, although they may subsequently have undergone gene amplification, over‐expression or gain‐of‐function mutations under pesticide selection.

The natural substrates of some efflux transporters and cytochrome P450s have been identified as plant defence compounds. Resistance to the synthetic quinolone antibiotics in the endophyte and opportunistic clinical pathogen *Stenotrophomonas maltophilia* is due to the over‐expression of an efflux pump normally induced in response to plant flavonoids such as phloretin during the colonisation of roots (Garcia‐Leon *et al.,* 2014). The neonicotinoid insecticides are analogues of nicotine, a defence compound produced by tobacco plants, and *Myzus persicae nicotianae* aphids, able to feed on tobacco plants due to overexpression of the nicotine‐metabolising enzyme *CYP6CY3*, are also resistant to neonicotinoid insecticides (Bass *et al.,* 2013).

Efflux transporters over‐expressed in azole fungicide‐adapted laboratory strains of *Fusarium graminearum* contribute to virulence: this may be through the removal of plant‐derived antifungal compounds, or through the secretion of fungal secondary metabolites (Abou Ammar *et al.,* 2013). ATP‐binding cassette (ABC) and major facilitator superfamily (MFS) transporter proteins confer self‐resistance of the plant pathogen *Cercospora nicotianae* to cercosporin, a toxin produced by the fungus itself to attack host plants (Beseli *et al.,* 2015). Therefore, both host and pathogen metabolites should be considered as potential natural substrates of transporters involved in pesticide resistance.

### (2) Pleiotropic effects

Where a pesticide has no known natural analogues, reduced pesticide sensitivity may be a pleiotropic effect of adaptations unrelated to toxin resistance. Fungal species with *CYP51A*, an additional *CYP51* paralogue with inducible upregulation, have intrinsic partial resistance to CYP51‐inhibiting fungicides including azoles (Fan *et al.,* 2013). The evolution of *CYP51A* pre‐dates anthropogenic fungicide use by 320–520 million years (Hawkins *et al*., 2014*b*) and no natural azole analogues are known; the evolution of *CYP51A* may instead be due to increased need for the sterol product of CYP51 under some conditions (Yan *et al.,* 2011), with the pleiotropic effect of allowing the fungus to withstand higher concentrations of CYP51 inhibitors. The target site of anti‐coagulant rodenticides, vitamin K epoxide reductase, is required for vitamin K recycling as well as blood clotting, and target site mutations conferring intrinsic warfarin resistance in *Mus spretus* may have originally evolved as an adaptation to a vitamin K‐deficient diet (Song *et al.,* 2011). Cummins *et al*. (2013) demonstrate the contribution of the *Alopecurus myosuroides* glutathione transferase *AmGSTF1* to multiple herbicide resistance, and suggest that glutathione transferases play a role in redox signalling in response to a range of abiotic stresses including drought and heat.

### (3) Neutral processes

Natural substrates or pleiotropic effects provide adaptive explanations for the selection of resistance mechanisms prior to anthropogenic pesticide exposure, resulting in the fixation of intrinsic resistance, or co‐optable pre‐resistance adaptations. However, where resistance is present at low frequencies as standing variation, this indicates that the resistant allele had not been selected to fixation prior to pesticide use. In some cases, this may be due to weak, patchy or fluctuating selection, resulting in incomplete selection or subsequent reversal, as reported by Mackie *et al*. (2010) for resistance to cadmium pollution in an aquatic worm. However, the maintenance of standing variation is generally assumed to be predominantly due to neutral or near‐neutral processes. This may apply to resistance‐conferring mutations with no selective advantage prior to pesticide use, but also to compensatory mutations or differences in genetic background that have epistatic interactions that become advantageous only when resistance evolves. For cryptic variation, with no observable fitness effects prior to pesticide selection (Gibson & Dworkin, 2004), standing variation is a product of mutation and genetic drift. For alleles that are actually deleterious in the absence of pesticide selection, standing variation equilibrates at the point of mutation–selection balance (Zhang & Hill, 2005).

## IV. DETECTION OF EVOLUTIONARY ORIGINS

In some cases, the source of a resistance allele may be detected directly, by finding resistant alleles in unselected populations, or by identifying the source species for interspecific gene transfer. In other cases, indirect detection is necessary, through selective signatures in genomes or population genetics. Distinguishing standing variation from *de novo* mutations through selective signatures has received considerable attention in recent years, and detection methods have been reviewed by Barrett & Schluter (2008) and Messer & Petrov (2013). Statistical methods continue to be developed (Ferrer‐Admetlla *et al.,* 2014), as well as methods taking advantage of increasing genomic data (Roesti *et al.,* 2014; Vatsiou, Bazin & Gaggiotti, 2016).

### (1) Summary of detection methods

#### (a) *Selective signatures*

Selection of a *de novo* mutation can be distinguished from selection from standing variation by ‘hitchhiking’ of linked genes, whereas a standing variant is likely to be found in multiple genetic backgrounds due to recombination prior to selection (Fig. 1). A hard selective sweep results from the rapid emergence of a mutation in a single genetic background, indicating a *de novo* mutation (Hermisson & Pennings, 2005). A soft sweep results from the selection of an allele in multiple genetic backgrounds, from standing genetic variation or recurrent *de novo* mutations (Pennings, Kryazhimskiy & Wakeley, 2014). Resistance alleles resulting from interspecific transfer may also carry signatures of selection, with hitchhiking by linked genes from the source species, if the hybridisation is rare and recent. In the case of HGT, a smaller region of DNA within a chromosome will have been transferred; a lack of synteny between haplotypes may provide a clearer genomic signature of gene transfer.

#### (b) *Unselected populations*

If populations were studied before selection took place, any resistance within an unexposed population would indicate standing variation. Alternatively, extant unselected populations may exist in other geographical regions. If historical samples are available, it may be possible to test for resistance retrospectively once the molecular basis of resistance is known (D'costa *et al.,* 2011). In the case of introgression or HGT, the resistant allele would instead be found first in the source species.

#### (c) *Phylogenetic dating*

The origin of a resistant allele can also be inferred by reconstructing the gene's phylogenetic tree and comparing it against the species phylogeny, to detect interspecific gene transfers or pre‐speciation standing variants (Fig. 2) (Colosimo *et al.,* 2005; Stern, 2013). For intraspecific origins, haplotype networks can be reconstructed. Mutations of recent origin will be found at the tips of branches on the haplotype network, whereas longer‐standing resistant genotypes will have accumulated further mutations, forming more extensive subtrees. Geographical information may also be included: resistance spread through migration will be found in the same haplotypic backgrounds as in the source population, whereas resistance emerging *in situ* will be found in haplotypic backgrounds common in the local sensitive population (Karasov *et al.,* 2010).

### (2) Applicability to resistance

The strong, recent selection for resistance, as well as common biological traits of pest species, make certain methods more suitable than others for many resistance studies.

Selective signatures may be confounded demographic factors, such as population bottlenecks due to founder effects if a pest has recently spread to a new region or jumped to a new host (Linde, Zala & Mcdonald, 2009). Comparison of linked and unlinked or closely and distantly linked genes is necessary to distinguish a selective sweep from a genome‐wide bottleneck. However, knowledge of linkage patterns is often lacking for non‐model species: whilst more genomes are being sequenced every year, not all next‐generation sequencing methods produce adequate assembly lengths. Furthermore, methods based on linkage assume regular recombination, which is not applicable in clonal populations, or for mitochondrial targets such as the QoI fungicide target site cytochrome *b* (Di Rago, Coppee & Colson, 1989). Detecting selective signatures is also more difficult for polygenic traits (Pritchard & Di Rienzo, 2010), such as metabolic resistance to some herbicides (Gaines *et al.,* 2014), but genome‐wide analyses are being developed to detect total selection acting across all loci associated with a trait (Berg & Coop, 2014).

Pre‐selection data are more likely to be available for resistance than many other systems, due to the recent and rapid nature of resistance evolution, as well as the practical importance of resistance risk assessments (Russell, 2004). By contrast, the analysis of populations in other geographical regions is more likely to be confounded by gene flow from selected populations (Barrett & Schluter, 2008), as pest species generally have good dispersal ability. It is also important that the unselected population has not been exposed to any compounds likely to show cross‐resistance, such as older compounds with the same mode of action.

Resistant alleles are sometimes detectable in pre‐exposure historical samples. Délye, Deulvot & Chauvel, 2013) detected herbicide‐resistant alleles in herbarium specimens. However, resistant alleles may have been present at very low frequencies before pesticide selection, so whilst their presence in a historical sample is conclusive, their absence is not. In the case of pathogens, a sample of host plant material may contain a larger population sample of the pathogen (Bearchell *et al.,* 2005; Yoshida, Sasaki & Kamoun, 2015), but detection limits depend on the methods used and DNA degradation in older samples.

In the case of interspecific gene transfer, direct detection of pre‐selection origins means finding the source species. A specific source may already be suspected, such as a herbicide‐resistant crop that may have interbred with wild relatives (Martins, Sun & Mallory‐Smith, 2015); otherwise, phylogenetic analysis of the transferred genetic region can be used to predict the source species (Fig. 2A).

For phylogenetic or haplotype approaches, the risk of homoplasy must be considered due to strong positive selection for resistance. Where resistance results from point mutations in an otherwise highly conserved gene, resistant alleles may form a false clade due to parallel evolution of the same *de novo* mutations. In order to be confident that a group of resistant alleles represents shared evolutionary origins (whether through lineage‐sorting or allele transfer) and not parallel mutations, it should be supported by synonymous substitutions or intron changes, or at least by substitutions shown to be phenotypically neutral with respect to resistance.

## V. PREVALENCE IN PESTICIDE RESISTANCE

### (1) Insecticides

The evolutionary origins of insecticide‐resistance alleles may include standing genetic variation, *de novo* mutation or, as has recently been demonstrated, adaptive introgression (Table 1).

**Table 1:** Summary of cases of pesticide resistance where evolutionary origins have been inferred

Organism	Pesticides	Resistance mechanism	Origins	References
Insects				
*Anopheles gambiae*	Pyrethroids	Target‐site mutation	*De novo,* multiple origins	Lynd *et al*. (2010)
*Anopheles gambiae*	OPs/CMs	Target‐site mutation	*De novo,* single origin	Weetman *et al*. (2015)
*Drosophila melanogaster*	OPs/CMs	Target‐site mutation	*De novo*, multiple origins	Karasov *et al*. (2010)
*Drosophila melanogaster*	DDT + others	Metabolic over‐expression	Standing variation	Catania *et al*. (2004)
*Plutella xylostella*	Diamides	Target‐site mutation	Standing variation	Troczka *et al*. (2012)
*Lucilia cuprina*	OPs	Metabolic mutation	*De novo* and standing variation	Hartley *et al*. (2006); Rose *et al*. (2011)
*Anopheles coluzzii*	Pyrethroids	Target‐site mutation	Adaptive introgression	Norris *et al*. (2015)
Weeds				
*Alopecurus myosuroides*	ACCase inhibitors	Target‐site mutation	Multiple independent origins; possibly standing genetic variation or *de novo* mutation	Délye *et al*. (2004, 2013); Menchari *et al*. (2006)
*Avena fatua*	ACCase inhibitors	Target‐site mutation	Multiple independent origins; possibly standing genetic variation	Mengistu, Messersmith & Christoffers (2003, 2005)
*Lolium rigidum*	ACCase inhibitors	Metabolic resistance	Standing genetic variation	Neve & Powles (2005a,*b*)
*Ipomoea purpurea*	Glyphosate	Unknown	Standing genetic variation	Baucom & Mauricio (2010); Kuester, Chang & Baucom (2015)
*Sorghum halepense*	Glyphosate	Non‐target site	Multiple independent origins	Fernández *et al*. (2013)
*Brassica rapa*	Glyphosate	Trans‐gene	Trans‐gene introgression	Warwick *et al*. (2008)
Pathogens				
*Plasmopara viticola*	QoIs	Target‐site mutation	*De novo*, two independent origins	Chen *et al*. (2007)
*Zymoseptoria tritici*	QoIs	Target‐site mutation	*De novo*, multiple parallel origins	Torriani *et al*. (2009)
*Zymoseptoria tritici*	Azoles	Target‐site mutations	*De novo*, sequential mutations	Cools & Fraaije (2013)
*Uncinula necator*	Azoles	Target‐site over‐expression (promoter insert)	*De novo*, single origin	Frenkel *et al*. (2015)
*Pyrenopeziza brassicae*	Azoles	Target‐site over‐expression (promoter insert)	Multiple origins	Carter *et al*. (2014)
*Aspergillus fumigatus* (clinical pathogen)	Azoles	Target‐site mutations and over‐expression	*De novo*, single origin for each haplotype	Camps *et al*. (2012)
*Botrytis cinerea*	Multiple fungicides	Target‐sites and enhanced efflux	Multiple resistance combinations: may indicate standing variation, multiple *de novo* origins or recombination	Fernández‐Ortuño *et al*. (2014)
*Rhynchosporium commune*	Azoles	Target‐site over‐expression (second paralogue)	Standing variation	Hawkins *et al*. (2014*b*)

ACCase, acetyl CoA carboxylase; CM, carbamate; DDT, dichlorodiphenyltrichloroethane; OP, organophosphate; QoI, quinone outside inhibitor.

Many early studies of insecticide resistance assumed *de novo* mutation as the source of resistance, particularly in the case of target‐site resistance where mutations were identified in functionally constrained receptors in the insect nervous system. More recent work has provided evidence supporting these initial assumptions with two examples: target‐site resistance to pyrethroids, and to organophosphates (OPs) and carbamates (CMs), in mosquitoes. Pyrethroid resistance in *Anopheles gambiae* is commonly associated with knockdown resistance (kdr) mutations (L1014F and L1014S) in the target site, the voltage‐gated sodium channel. Lynd *et al*. (2010) employed haplotype diversity analyses to investigate genetic variation around the kdr locus in *An. gambiae s.s*. A loss of genetic diversity was associated with the kdr mutations, with the L1014F kdr mutation exhibiting an especially pronounced footprint of a hard selective sweep, strongly suggestive of evolution by *de novo* mutation.

The G119S substitution in the gene encoding acetylcholinesterase 1 (*Ace‐1*) is widespread in mosquitoes and confers resistance to OPs and CMs. Weetman *et al*. (2015) identified a single *Ace‐1* resistant haplotype, with reduced nucleotide diversity and high linkage disequilibrium compared to *Ace‐1* wild‐type haplotypes, again providing strong evidence of a recent *de novo* origin rather than selection from standing genetic variation.

In contrast to the classical hard‐sweep scenarios described above, investigation of OP/CM resistance in *Drosophila melanogaster* has provided an example of adaptation from multiple *de novo* mutations producing a soft selective sweep (Karasov *et al.,* 2010). Four different point mutations, I161V, G265A, F330Y and G368A, in the *Ace* gene lead to OP and CM resistance in *D. melanogaster*, and all resistant alleles are strongly deleterious in the absence of insecticide. By sequencing the *Ace‐1* gene in a large number of resistant and susceptible fly strains, Karasov *et al*. (2010) provided evidence of multi‐step adaptation, with resistant haplotypes comprising three SNPs arising from *de novo* mutations just a few years after the introduction of OPs and CMs. Significantly, the authors found that *Ace‐1* mutations arose repeatedly, even within the same continent, demonstrating a far higher rate of adaptive mutation in fruit flies than previously envisaged, and indicating that in some cases, adaptive potential from *de novo* mutations may be higher than that predicted from standing variation levels.

In other cases, the rapid emergence of insecticide resistance is thought to reflect evolution from standing genetic variation. Resistance to the new diamide insecticides evolved in the diamondback moth, *Plutella xylostella*, within just two years of use. Resistance was found to be associated with an amino acid substitution, G4946E, in the target site, the ryanodine receptor (RyR) (Troczka *et al.,* 2012). Interestingly, the nucleotide changes that result in the G4946E substitution in two geographically separated resistant strains were different and susceptible strains also show synonymous polymorphism at this codon. The *P. xylostella* genome is known to be extremely polymorphic (You *et al.,* 2013) and the sequence diversity observed in the RyR of resistant and susceptible strains, combined with the extremely rapid emergence of resistance, is suggestive of selection from standing variation.

Population‐genetic studies examining the evolution of insecticide resistance in *D. melanogaster* have also suggested that adaptation from standing genetic variation can occur in the case of metabolic resistance (Catania *et al.,* 2004). Resistance to dichlorodiphenyltrichloroethane (DDT) and several other insecticide classes in *D. melanogaster* is conferred by enhanced expression of the P450 cyp6g1 resulting from the insertion of an Accord transposable element in the promotor of the gene encoding this enzyme. Investigation of the distribution of the Accord element in 673 *D. melanogaster* lines from 34 world‐wide populations revealed a much narrower selective sweep around the insertion than would be expected for a *de novo* mutation under strong selection, suggesting that this insertion may pre‐date the use of DDT (Catania *et al.,* 2004).

Evolution of insecticide resistance from standing genetic variation and by *de novo* mutation are not necessarily mutually exclusive. Work on the sheep blowfly, *Lucilia cuprina*, provides an example of both origins in resistance to OPs (Hartley *et al.,* 2006; Rose *et al.,* 2011). Resistance to the OP insecticides diazinon and malathion in this species is conferred by two independent mutations in esterase E3 encoded by the *Lcα7* gene. The G137N substitution confers resistance to diazinon while alterations at a second site, W251L/S/T, confers resistance to malathion. Analysis of *Lcα7* in Australasian strains of *L. cuprina*, including pinned specimens collected prior to the introduction of OP insecticides, has suggested that two incomplete soft sweeps occurred at this locus. Several resistance mutations at position 251 were observed in pinned specimens, providing unambiguous evidence of genetic variation existing before the first use of OPs. Conversely, the G137N mutation was only identified in post‐OP samples, suggesting that diazinon resistance may have evolved later by *de novo* mutation. The different origins for two resistance mutations in the same gene is intriguing and is likely related to their relative fitness cost, with mutation of position 137 carrying a much greater fitness penalty than substitutions at 251 (Hartley *et al.,* 2006). Indeed, the G137N substitution may only have risen to high frequency because it confers much greater resistance to the more widely used diazinon insecticide and because of the proliferation of a compensatory mutation at a separate *Modifier* locus (Hartley *et al.,* 2006).

Beyond *de novo* mutation and standing genetic variation, recent work has highlighted a third potential evolutionary origin of insecticide resistance alleles, through adaptive introgression (Norris *et al.,* 2015). The malaria mosquitoes *An. gambiae* and *An. coluzzii* are sympatric across much of sub‐Saharan Africa with hybridisation occurring at varying frequencies across the range of the two species, although hybrids typically suffer a fitness disadvantage and the species generally show assortive mating. This results in a degree of reproductive isolation, and the two species differ in their level of resistance to insecticides, with several insecticide‐resistance alleles in *An. gambiae* located within a genomic island of divergence on Chromosome 2. Norris *et al*. (2015) carried out a longitudinal population‐genetic study of the two mosquito species in Selinkenyi, Mali, and demonstrated that during a breakdown in assortive mating in 2006, *An. coluzzi* inherited the genomic island from *An. gambiae*, gaining several insecticide‐resistance alleles including the kdr mutation L1014F that was previously absent from *An. coluzzii*. Hybrid individuals carrying the resistant alleles were strongly selected for, and backcrossed with parental populations. The introgression event was coincident with the start of a major vector control initiative in Mali using insecticide‐treated bed nets, suggesting that a change in the fitness landscape allowed the genomic island of divergence to cross the reproductive barrier between the two species by favouring the survival of the normally less fit hybrids (Norris *et al.,* 2015).

### (2) Herbicides

The molecular and biochemical mechanisms conferring resistance to herbicides have been well established, particularly for acetyl‐CoA carboxylase (ACCase), acetolactate synthase (ALS) and 5‐enolpyruvylshikimate‐3‐phosphate (EPSPS) inhibiting herbicides (Powles & Yu, 2010). For all three modes of action, target‐site and non‐target‐site mechanisms of resistance have been documented, with prevailing evidence suggesting that non‐target‐site metabolic resistance is the predominant mechanism. Target‐site and non‐target‐site mechanisms are not mutually exclusive, as both can be found within the same population, and even occur within the same individual (Yu & Powles, 2014). There have been relatively few studies that have employed population‐genetic and genomic techniques to address questions related to the evolutionary origins of the mutations underpinning these adaptations (Table 1). This is, at least in part, due to the lack of development of genomic resources for major weed species, and, in the case of non‐target‐site resistance, to scant knowledge of the genetic basis of resistance, although this situation is now being addressed (Cummins *et al.,* 2013; Gaines *et al.,* 2014). The available evidence largely supports the phenomenon of multiple evolutionary origins of resistance (Délye *et al.,* 2013), implying that the signature of soft selective sweeps would be detected for herbicide resistance.

For ACCase‐ (Kaundun, 2014) and ALS‐inhibiting (Yu & Powles, 2014) herbicides, multiple SNPs have been documented, resulting in a number of different amino acid substitutions that confer resistance to these herbicides, across and within species and individuals. The existence and global distribution of these mutations in a number of species also suggests multiple independent origins of resistance at a broad scale. Délye *et al*. (2004) used phylogenetic analysis of the ACCase sequence in nine populations of the grass weed, *A. myosuroides* to demonstrate that there had been four and six independent origins of the L1781I and N2041I substitutions, respectively, across nine populations of the species. Further ACCase sequencing over a larger set of *A. myosuroides* populations in France identified L1781, N2041 and A2096 haplotypes differing by up to eight, 11 and 10 mutations, respectively (Menchari *et al.,* 2006), further supporting multiple independent origins. These studies did not explore signatures of selection around resistance‐conferring loci to infer whether mutations arose from standing genetic variation or by *de novo* mutation. However, Délye *et al*. (2013) reported the existence of the L1781 mutation in a herbarium sample of *A. myosuroides* collected in 1881, almost 100 years prior to the introduction of these herbicides. In *Avena fatua*, Mengistu *et al*. (2003) showed that ACCase‐resistant individuals were present in populations collected prior to herbicide use, and subsequent investigation of genetic variation in a series of resistant populations indicated multiple origins of resistance (Mengistu *et al.,* 2005).

For non‐target‐site resistance to ACCase herbicides, evolution of resistance from standing genetic variation has been strongly implicated by selection experiments in the greenhouse (Neve & Powles, 2005a,*b*) and in the field (Manalil *et al.,* 2011). Neve & Powles (2005a) found mean phenotypic frequencies of resistance (survival at field recommended rates) of 0.4% across 31 populations of *Lolium rigidum* with no previous history of herbicide exposure. Combining these results with *in vitro* assays, they demonstrated that high‐frequency resistance within standing genetic variation in these populations was due to non‐target‐site resistance mechanisms.

For glyphosate resistance, a number of studies have coupled population‐genetics and phenotypic data sets from population samples collected prior to the introduction of glyphosate, suggesting that multiple independent evolutionary origins are due to standing genetic variation. Using simple‐sequence repeat (SSR) markers, Fernández *et al*. (2013) showed that glyphosate‐resistant *Sorghum halepense* populations have independently evolved glyphosate resistance. Okada *et al*. (2013) demonstrated that glyphosate resistance in *Conyza canadensis* populations across California has evolved *via* multiple independent evolutionary origins, with subsequent dispersal after resistance has evolved. Baucom & Mauricio (2010) grew *Ipomoea purpurea* from preserved seed collected in the 1980s, prior to the widespread introduction of glyphosate in the mid‐1990s, and compared levels of glyphosate sensitivity to a modern population. They found variation in glyphosate resistance in the historical as well as the modern population, indicating that glyphosate resistance was present in standing variation prior to herbicide selection. Kuester *et al*. (2015) analysed neutral genetic markers in *I. purpurea* and demonstrated that populations experienced gene flow pre‐glyphosate usage in the USA but glyphosate resistance then evolved independently in multiple populations, and concluded that this independent evolution of resistance was through selection from the standing variation demonstrated by Baucom & Mauricio (2010).

Herbicide‐resistance genes have also been reported to originate through processes of hybridisation and introgression between crops and related weed species, although there is little evidence that this is a major source of field‐evolved herbicide resistance. There has been much debate about the potential for transfer of resistance genes between genetically modified crops and closely related wild, weedy relatives through processes of hybridisation and subsequent introgression (see review by Kwit *et al.,* 2011). Warwick *et al*. (2008) demonstrated that herbicide‐resistance genes from transgenic *Brassica napus* crops were introduced into weedy *B. rapa* populations and then maintained for six years in these weedy populations in the absence of herbicide selection and despite the fitness cost of hybridisation. Watrud *et al*. (2004) reported gene flow between genetically modified (GM) *Agrostis stolonifera* and weedy populations of the same species and Reichman *et al*. (2006) followed this report by demonstrating that individuals with the transgene had established in natural habitats. However, in most cases, herbicide‐resistance has evolved independently within weed species, due to the strong selection imposed by repeated use of the single herbicide to which the GM crop is resistant (Powles, 2008).

Although much work has been focussed on the hybridisation of genetically modified organisms (GMOs) and their wild relatives, allele transfer through hybridisation may also occur between conventionally bred herbicide‐resistant crops and wild relatives (Mallory‐Smith & Olguin, 2011), or between related weed species [see Gaines *et al.,* 2012 and citations therein]. Martins *et al*. (2015) detected the imazimox‐resistant Imi1 allele from imazimox‐resistant wheat in wheat × jointed goatgrass (*Aegilops cylindrica*) hybrids and backcrossed plants. Gaines *et al*. (2012) demonstrated through field and greenhouse crosses that inter‐specific hybridisation can transfer glyphosate resistance, conferred by EPSPS gene amplification, from *Amaranthus palmeri* to other weedy *Amaranthus* species.

### (3) Fungicides

Most studies so far that have explicitly considered the evolutionary origins of fungicide‐resistant alleles have found evidence for *de novo* mutations as the main source of resistance (Table 1).

Resistance to QoI fungicide is commonly conferred by the G143A substitution in mitochondrially encoded target site cytochrome *b*. Chen *et al*. (2007) analysed the cytochrome *b* haplotypes in the grapevine powdery mildew *Plasmopara viticola*, finding that G143A is present in two different haplotypic backgrounds, with the reconstructed haplotype network indicating parallel *de novo* mutations. Torriani *et al*. (2009) analysed cytochrome *b* haplotypes in the wheat pathogen *Zymoseptoria tritici*. The G143A substitution was present in 24 different haplotypes, with phylogenetic analysis indicating four independent *de novo* origins. Estep *et al*. (2015) subsequently investigated cytochrome *b* haplotypes in *Z. tritici* populations from North America, finding G143A present in several haplotypic backgrounds, with phylogenetic analysis supporting multiple parallel origins. However, they also report evidence of reduced mitochondrial genetic diversity among isolates with G143A, so the overall picture is of a selective sweep intermediate between a soft and hard sweep, following a small number of parallel *de novo* mutations.

Resistance to azole fungicides in *Z. tritici* is due to multiple mutations in the target‐site‐encoding gene *CYP51*. Earlier isolates had only one or few mutations, conferring low levels of resistance, but more recent isolates have accumulated more mutations, building up higher levels of resistance (Cools *et al.,* 2006; Cools & Fraaije, 2013). The sequential accumulation of *CYP51* mutations indicates *de novo* origins, with more recent mutations appearing in genetic backgrounds already including previously selected mutations. For example, the recently emerged mutation S524T, conferring reduced sensitivity to the recently introduced fungicide prothioconazole, is not found as a single mutation in a wild‐type background, only in combination with other mutations such as substitutions or deletions at codons 459–461. Site‐directed mutagenesis studies have shown that *Z. tritici CYP51* with only the S524T substitution in a wild‐type background would be adaptive under prothioconazole selection, conferring reduced sensitivity to prothioconazole whilst retaining enzyme function (Cools *et al.,* 2011); therefore its occurrence only in combination with other mutations and not in a wild‐type background does not reflect functional constraints, but is a result of its *de novo* origin after other mutations had been selected to higher frequencies in the population. However, *Z. tritici* populations continue to contain a mixture of many different *CYP51* haplotypes (Curvers *et al.,* 2014; Wieczorek *et al.,* 2015), with no single allele being selected to fixation, and substitutions such as I381V, V136A and S524T appear to have arisen multiple times through parallel or convergent evolution in different backgrounds. Thus *Z. tritici CYP51* is likely only to have undergone soft selective sweeps despite the *de novo* mutations.

In the grapevine powdery mildew *Uncinula necator*, azole resistance is conferred by *CYP51* target site mutations, or *CYP51* overexpression due to a promoter insert. All *CYP51* overexpressing isolates sequenced so far carry a linked synonymous mutation in the coding region (Frenkel *et al.,* 2015), indicating a hard selective sweep following a *de novo* mutation in a single genetic background. By contrast, in the oilseed rape pathogen *Pyrenopeziza brassicae*, *CYP51* over‐expression is due to one of several promoter inserts of different lengths (Carter *et al.,* 2014), suggesting that *CYP51* over‐expression has multiple origins, which would result in a softer selective sweep whether from standing variation or multiple *de novo* origins. In the opportunistic clinical pathogen *Aspergillus fumigatus*, a 34‐base pair tandem repeat in the promoter region is found in combination with the L98H mutation (Mellado *et al.,* 2007), whereas a 46‐base pair tandem repeat is found in combination with Y121F/T298A, and isolates with TR_34_/L98H also have reduced genetic diversity overall as measured by microsatellites and cell surface protein types (Camps *et al.,* 2012), indicating a single *de novo* origin for each.

Fernández‐Ortuño *et al*. (2014) investigated multi‐fungicide‐resistant strains of the soft fruit pathogen *Botrytis cinerea*. They sequenced five target‐site‐encoding genes and an efflux transporter transcription factor for isolates resistant to seven different classes of fungicides. No sequenced resistant isolates had identical genotypes across the six genes, leading the authors to conclude multiple independent origins of multi‐fungicide‐resistance. However, it is not clear whether the multiple resistance originates from independent parallel mutations, or independent recombination events between single‐fungicide‐resistant strains, and therefore it is not possible to infer whether those mutations occurred *de novo*, or whether at least the single‐resistance mutations were selected from standing variation.

One case of fungicide resistance selected from standing variation was reported by Hawkins *et al*. (2014*b*). In the barley pathogen *Rhynchosporium commune*, isolates possessing *CYP51A*, an additional paralogue of the target‐site‐encoding gene *CYP51*, show reduced azole sensitivity and have increased in frequency under azole selection since the 1980s, but phylogenetic analysis shows that *CYP51A* diverged from the other *CYP51* paralogue, *CYP51B*, around 400 million years ago. In other species with *CYP51A*, its presence appears to be fixed, conferring intrinsic partial resistance to azoles (Fan *et al.,* 2013), and possibly conferring some pre‐disposition for the evolution of further resistance, as mutations and overexpression occur predominantly in that paralogue where present (e.g. Mellado *et al.,* 2007), perhaps because the second paralogue is less affected by functional constraints than the constitutively expressed ‘workhorse’ *CYP51B* (Hawkins *et al*., 2014*a*). *CYP51A* expression is induced by azole exposure (Fan *et al.,* 2013; Hawkins *et al*., 2014*b*), so paralogue presence or mutations could be seen as cryptic variation, with phenocopy induction by fungicides, although its as yet unidentified pre‐azole function is likely to involve induced expression under certain other conditions too.

There are currently no known cases of fungicide resistance originating from adaptive introgression or horizontal transfer. However, evidence of introgression has been found in fungal genomes (Neafsey *et al.,* 2010), and adaptive introgression has played a role in fungal adaptation to anthropogenic environments such as the domestication of wine yeasts (Marsit *et al.,* 2015). Evidence of HGT has also been found in fungal genomes (Cheeseman *et al.,* 2014), and it has played a role in the evolution of a plant pathogenic fungus, *Pyrenophora tritici‐repentis*, which emerged in the mid‐20th century after acquiring the *ToxA* toxin‐encoding gene from *Stagonospora nodorum* (Friesen *et al.,* 2006), with further studies inding that the *P. tritici‐repentis* genome also contains genes transferred from bacteria (Sun *et al.,* 2013). Therefore, adaptive introgression and HGT are potential sources of fungal adaptation.

## VI. CONTRASTS AMONG PESTICIDE GROUPS

From studies reported to date, the emerging picture is that herbicide resistance evolves largely by selection from standing genetic variation, especially in the case of metabolic resistance with fewer cases of target‐site resistance selected from standing variation. Insecticide resistance has evolved variously through selection from standing variation and *de novo* mutations, with both metabolic and target‐site resistance mechanisms commonly occurring. Fungicide resistance has evolved predominantly through *de novo* mutations, with mostly target‐site resistance.

Various *ad hoc* reasons may be suggested to explain these differences: perhaps plants have greater standing variation in order to metabolise other toxins; perhaps pathogens have higher *de novo* mutation rates to engage in host–pathogen arms races. However, individual population‐genetic parameters such as mutation rate or genetic diversity should be considered in the context of a broader evolutionary model of when resistance will evolve from standing variation, *de novo* mutations, a combination of both, or not at all.

**Figure 3:**
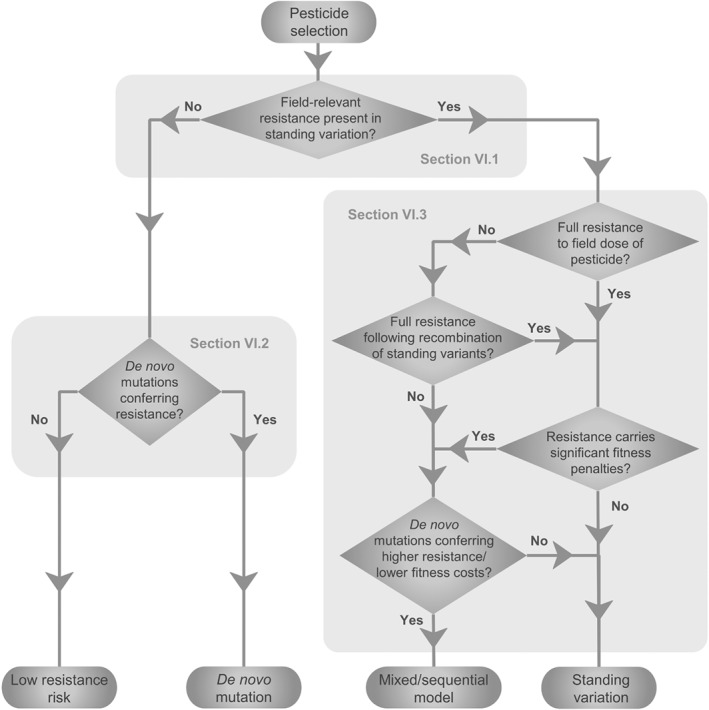
Proposed model for determining whether resistance will evolve from standing variation or *de novo* mutations for a given pest–pesticide system. Factors influencing the outcome of each question are discussed in the text sections referred to in the grey text.

Our proposed model schematic is shown in Fig. 3. Where resistance is present in standing variation, those variants will be selected, since they are already present, usually in more than one individual, and can therefore emerge more rapidly than *de novo* mutations (see Section II.2). If the initial population lacks resistant alleles, then *de novo* mutations will be selected when and if they arise. However, if standing variation confers only partial resistance relative to field doses, those variants will be selected initially, but subsequent *de novo* mutations that confer higher resistance will then be selected. A similar scenario would be possible if the initially selected standing variants carried higher fitness penalties than potential *de novo* mutations, although this would make them less likely to persist prior to pesticide selection unless the fitness cost was recessive (and unlikely to be homozygous at pre‐selection frequencies) or conditional (such as an inducibly expressed detoxification pathway). The overall risk of resistance evolving reflects the combined risk from standing variation and *de novo* mutations.

Therefore, the outcome will depend on levels of standing variation, rates of *de novo* mutation, and the relative resistance and fitness conferred by each at field doses of the pesticide. The effect of these three key factors is summarised in Fig. 3, and the pathogen‐ and pesticide‐related parameters affecting the outcomes in different pesticide classes are discussed below.

### (1) Standing variation levels

Observed patterns of evolution imply that levels of standing variation are highest in weeds, then insect pests, then fungal pathogens.

A model for evolution from standing variation proposed by Hermisson & Pennings (2005) comprises terms relating to effective population size, mutation rate, the fitness coefficients before and after the change in selection, and the dominance of those fitness effects. Mutation rate, initial fitness and effective population size determine variation levels at mutation–selection–drift balance. Mutation rate refers to relevant mutations, and will therefore be affected by the likelihood and complexity of the resistance mechanism in question as well as overall mutation rate.

Experimental evolutionary studies have shown that selection from standing variation can drive adaptations in sexually outcrossing species, where recombination contributes to genetic variation, forming new combinations of existing alleles (Burke *et al.,* 2014). Therefore, higher standing variation might be expected in outcrossing weed species, compared to many polycyclic fungal pathogens and some insects such as aphids, which undergo repeated clonal reproduction during a crop growing season.

Alternatively, weeds may maintain higher levels of standing variation due to lower rates of loss of diversity. Standing variation reflects mutation–selection–drift balance; reduced mutation rates would be expected to reduce *de novo* mutation, so a species with less standing variation but high rates of *de novo* mutation may be experiencing stronger purifying selection. This could reflect more stringent selection overall, or higher fitness costs of resistance mutations, but there is little evidence of either at present.

Loss of diversity can also result from demographic factors. Plant pathogens and phytophagous insects have undergone past population bottlenecks during host jumps (Groman & Pellmyr, 2000; Messina, Mendenhall & Jones, 2009); whereas weeds, which act as competitors rather than parasites to crop plants (with very few exceptions), are not host dependent or host specialised to the same extent. However, the high and stable host populations provided by agricultural crops mean that once a pest or pathogen has colonised a crop species, they will experience fewer bottlenecks than relatives on wild plants (Stukenbrock *et al.,* 2007), and there is more evidence of bottlenecks from geographic range shifts (Munkacsi, Stoxen & May, 2008; Linde *et al.,* 2009), which would be expected to affect all pest groups.

Population‐genetic factors affect the overall level of standing genetic variation in a population, but other factors may specifically affect variation conferring resistance. Plants may have more relevant standing variation, especially for metabolic resistance, due to a wider pre‐existing metabolic range. This is often explained on the grounds that plants are non‐motile, and therefore must rely on detoxification rather than avoidance. However, fungal pathogens, with the lowest propensity to evolve pesticide resistance from standing variation in metabolic detoxification, are also sessile; and whilst insects are capable of chemotaxis and host choice, they have evolved metabolic adaptations to defence compounds produced by their own host plant. Fungal pathogens cannot move to find a suitable host, instead relying on the production of large numbers of spores so that some will land on the right plant. Therefore, the contrast between plant weeds and fungal pathogens is due not to differences in mobility, but perhaps to the types of eco‐evolutionary differences sometimes simplified to *r*/*K* selection (Reznick, Bryant & Bashey, 2002) or competitor–stress tolerator–ruderal (CSR) strategies (Grime, 1988). Whilst weed and pest species tend to be *r*‐strategists compared to other species within their taxa, comparisons among taxa would suggest that micro‐organisms are more extreme *r*‐strategists than macro‐flora. Therefore, plants may have evolved a higher degree of phenotypic plasticity so that an individual can cope in a range of surroundings, while pathogens rely more on the extreme over‐production of propagules for dispersal so that some will fall in favourable conditions – a strategy which may also favour higher rates of *de novo* mutation. This may be because pathogens must repeatedly colonise new host plants and new growth of host plants, such as new leaf layers, whereas weed seed banks may accumulate leading to a more competitive situation.

### (2) *De novo* mutation rates

The main parameters in models of evolution by *de novo* mutation are mutation rate, and selection coefficient. Whilst the generation of standing variation also depends on the rate of relevant mutations, this is as a component of longer‐term mutation–selection–drift balance such that a lower relevant mutational supply could be balanced out by lower rates of variant loss, whereas for rapid evolution by *de novo* mutations, a high enough rate of relevant mutation is crucial.

Fungal pathogens generally have shorter generation times than weeds, with most species going through multiple generations per growing season. Therefore, even with a similar mutation rate per generation, they would have a higher mutation rate per unit time. Furthermore, pathogens tend to have larger populations given their smaller size, so mutation rates per individual would be multiplied by a greater population size to give a higher total mutational supply (Messer & Petrov, 2013).

The emergence of mutations may be faster in haploid organisms, including most plant pathogens, since a single mutation is subject to selection immediately; in diploid or polyploid organisms, including weeds, dominant alleles are subject to selection immediately, but recessive alleles must first reach homozygosity without being lost through genetic drift (Orr & Betancourt, 2001). Rust fungi are diploid, and have been considered to be at low risk of evolving fungicide resistance, although their low resistance risk is partly due to mutational constraints such as intron splice sites (Oliver, 2014), and some cases of fungicide resistance due to target‐site mutations have now been reported in rust fungi (Schmitz *et al.,* 2014).

The influence of ploidy on resistance evolution also needs to be considered for several economically important arthropods such as whiteflies, thrips and spider‐mites. These species are haplodiploid and this is predicted to accelerate resistance development as a consequence of novel mutations being exposed directly to selection, irrespective of dominance, in hemizygous males. However, this prediction is based on the assumption that the resistance of R males is equivalent to RR females, which is valid for some species and insecticides but not all (Carrière, 2003).

The potential for a *de novo* mutation from a single origin to spread through a population also depends on migration rates (McDonald & Linde, 2002). Fungal ascospores can carry resistance alleles long distances (Fraaije *et al.,* 2005; Torriani *et al.,* 2009). Insecticide resistance can also spread through insect migration (Raymond *et al.,* 1991), although in other cases resistance has been selected independently in geographically isolated populations (Troczka *et al.,* 2012). A study of gene flow and herbicide resistance in the weed *Ipomoae purpura* concluded that historical gene flow had led to shared standing variation, but lower gene flow within the timeframe of glyphosate use meant that resistance had evolved independently in local populations (Kuester *et al.,* 2015).

### (3) Relative resistance levels

The selection of resistance depends not just on the presence or absence of standing variation or *de novo* mutations, but on the levels of resistance conferred by each variant, relative to the selecting dose of the pesticide. When the dose is sufficiently low for standing variants to confer resistance, those variants will be selected; when the dose is beyond the sensitivity range of the standing variation, then more highly resistant *de novo* mutations will be selected. If doses are lower than the resistance possible from small‐effect alleles present as standing variation, those alleles will be selected rapidly before any *de novo* mutations emerge, and there will then be no further selection for new mutations unless the dose rate is subsequently increased beyond the range of polygenic resistance. If dose rates are higher, small‐effect alleles offer little selective advantage as the pesticide will still be lethal to individuals carrying such alleles, but once highly resistant *de novo* mutations occur, those will be selected.

The importance of the nature of selection, as well as the population genetics and overall mutation rate of the target organism, is demonstrated by cases of evolution from standing variation and *de novo* mutations occurring within the same species. In the clinical bacterial pathogen *Streptococcus pneumoniae*, drug resistance evolved from *de novo* mutations, but standing variation in serotype led to vaccine escape (Croucher *et al.,* 2014). In *Drosophila melanogaster*, target‐site resistance to OPs resulted from multiple *de novo* mutations (Karasov *et al.,* 2010), whereas metabolic resistance to DDT was selected from standing variation (Catania *et al.,* 2004).

As discussed in Section II.2, *de novo* mutations, starting from a lower frequency than standing variants, need to be under stronger positive selection to be likely to emerge: this means highly resistant alleles are more likely than partial‐resistance alleles to emerge and persist following *de novo* mutations. Conversely, metabolic resistance may be more likely than target‐site resistance to be present as standing variation: pesticides target essential genes, which may be more conserved due to functional constraints than secondary metabolism and detoxification pathways which may be more variable to allow adaptation to various naturally occurring toxins.

This explanation would be testable by analysing baseline sensitivity distributions for herbicides, fungicides and insecticides, with the prediction that whilst standing variation may be present across all groups, the highest levels of fungicide resistance would all lie well below field dose rates, whereas the most herbicide‐resistant plants would be at levels closer to field resistance. However, this would require genuinely unexposed populations of a range of species. Alternatively, experimental evolution approaches could be used, using both a mixed starting population and mutagenesis if required, and comparing the results of selection with high and low doses of a pesticide.

The different resistance levels likely to persist as standing variation or to emerge *de novo* have practical implications for the optimal pesticide dose rates for resistance management. The long‐standing advice to use full dose rates applies in cases where standing variation includes individuals that would be selected by lower doses but controlled at a full dose. However, where the greater risk is highly resistant *de novo* mutations, higher doses will exert stronger selective pressure for the emergence of such mutations and therefore lower doses would slow the evolution of resistance (Van Den Bosch *et al.,* 2011). Where both types of resistance evolution are possible, a ‘revolving dose strategy’ alternating low and high doses may be optimal (Gardner, Gressel & Mangel, 1998).

In some cases, the model predicts a series of resistance mechanisms evolving over time. When a population is exposed to a pesticide, initially selection from standing variation would result in rapid but smaller shifts in sensitivity, followed by the selection of more resistant *de novo* mutations as they arise. A classic example of multiple adaptive steps occurring in rapid succession is the evolution of insecticide resistance in *D. melanogaster* mediated by the overproduction of the P450 Cyp6g1. This example of allelic succession at the *Cyp6G1* locus has occurred over a 70‐year period with each step resulting in increasing resistance (Schmidt *et al.,* 2010). However, sequential increases in resistance could also result from recombination of multiple quantitative resistance alleles; or from the accumulation of multiple *de novo* mutations, as seen with *CYP51* mutations conferring azole resistance in *Zymoseptoria tritici* (Cools & Fraaije, 2013). Furthermore, where *de novo* mutation rates are very high, the waiting time may be negligible, with selection operating on standing and *de novo* mutations simultaneously, selecting whichever has the optimal balance of resistance to field doses and low fitness penalties. More research is needed to test the model proposed here: quantifying the relevant parameters (diversity levels, mutation rates, baseline sensitivity distributions, temporal processes in resistance evolution) in field populations, defining mutant‐selective windows for pest species in the laboratory, and testing various scenarios through experimental evolution. More explicit studies of the evolutionary origins of resistance are also needed across the three groups to increase the sample size for each and see whether the pattern of standing variation in weeds, mixed origins in insects, and *de novo* mutation in fungi actually holds across a greater number of species and modes of action. Further analysis of differences within, as well as between, the three groups may also be informative; for example, is polygenic resistance selected from standing variation more common in outcrossing insects, and *de novo* emergence of major metabolic or target‐site resistance more common in insects with clonal reproduction?

## VII. FUTURE PROSPECTS

Ever‐increasing cases of resistance, combined with regulatory threats to some current chemicals and a slowing pipeline of new products, is leading to a shrinking toolkit for the control of many key pests. Therefore, it is vital to understand the processes involved in the evolution of resistance, including its evolutionary origins and the implications for resistance risk and management, in order to prolong the useful life of existing compounds.

Furthermore, many pest species under selection for resistance have large populations, short generation times and strong, defined selective pressures, making them suitable models for investigating wider, fundamental questions regarding rapid adaptation to novel selective pressures. This may include questions regarding the importance of standing variation and *de novo* mutations; the reasons for their differing prevalence in different systems; the role of functional constraints in determining which adaptive options are available to an evolving population; and the impacts of demography on adaptive potential.

The methods summarised here for detecting the evolutionary origins of adaptations to new selective pressures should be applied to more cases of pesticide resistance, to assess the extent to which the observations presented here from the studies carried out to date can be generalised. This will be aided by the increasing availability of genomes for pests as well as model species (I5K Consortium, 2013; Peng *et al.,* 2014; Pedro *et al.,* 2016). New tools will also allow wider investigations into the role of epigenetics in adaptation (Charlesworth, Barton & Charlesworth, 2017).

Many of the implications for resistance risk and management also apply to non‐chemical control measures, such as resistant plant varieties, semiochemical lures and repellents, and potential future biotechnological approaches including gene silencing or gene drives (Unckless, Clark & Messer, 2017). When assessing the risk of a pest evolving to overcome a control measure, it is important to consider each possible source of resistance: a lack of resistance in the current field population does not exclude resistance risk from *de novo* mutations, whereas a requirement for multiple mutations or complex resistance mechanisms that are unlikely to arise *de novo* does not exclude resistance risk from standing variation or interspecific transfer. Understanding the probable sources of resistance before a control measure is deployed would increase the chances of successful pro‐active resistance monitoring and management.

## VIII. CONCLUSIONS

(1) Resistance may originate by *de novo* mutations occurring once the pesticide is being used; selection from pre‐existing standing genetic variation; or from resistant species *via* hybridisation or HGT.

(2) The origins of pesticide resistance have practical implications for designing appropriate resistance risk assessments; the reliability of molecular detection methods across different populations and species; and the most effective measures for managing or containing resistance.

(3) Pests may be pre‐adapted to pesticides due to exposure to natural inhibitors such as host defence compounds or pathogen toxins, or as a pleiotropic effect of traits unrelated to chemical resistance, whereas standing variation may accumulate through neutral processes.

(4) The origins of a resistant allele may be detected through the selective signatures of hard or soft sweeps; direct detection of resistance in unexposed populations; phylogenetic reconstruction of the origins of an allele; or haplotype mapping of the genetic backgrounds in which resistance is found.

(5) In cases that have been investigated so far, herbicide resistance is often due to selection of polygenic metabolic resistance from standing variation; fungicide resistance is most commonly due to *de novo* target‐site mutations; and insecticide resistance has cases of both *de novo* mutations and selection from standing variation, in target‐site and major metabolic enzyme‐encoding genes.

(6) Allele transfer, whether horizontal or by hybridisation, is rarer in all three groups (weeds, insect pests and fungal plant pathogens) than HGT in the spread of antibiotic resistance in bacteria. Herbicide‐resistant weeds are more likely to arise in genetically modified herbicide‐resistant crops by convergent or parallel evolution of resistance within the weed species than by transgene escape.

(7) Differences between pesticide groups may reflect demographic and epidemiological factors, mutation rates, metabolic repertoire, or pesticide exposure rates relative to resistance factors.

(8) Pesticide resistance provides many examples of rapid, recent evolution in action, which can address fundamental questions regarding the origins of adaptations to novel selective pressures.
